# The Hippo-Salvador signaling pathway regulates renal tubulointerstitial fibrosis

**DOI:** 10.1038/srep31931

**Published:** 2016-08-23

**Authors:** Eunjeong Seo, Wan-Young Kim, Jeongmi Hur, Hanbyul Kim, Sun Ah Nam, Arum Choi, Yu-Mi Kim, Sang Hee Park, Chaeuk Chung, Jin Kim, Soohong Min, Seung-Jae Myung, Dae-Sik Lim, Yong Kyun Kim

**Affiliations:** 1 National Creative Research Initiatives Center, Department of Biological Sciences, Korea Advanced Institute of Science and Technology, Daejeon, Korea; 2 Biomedical Research Center, Asan Institute for Life Sciences, Seoul, Korea; 3 Department of Anatomy and Cell Death Disease Research Center, College of Medicine, The Catholic University of Korea, Seoul, Korea; 4 Institute of Clinical Medicine Research of Bucheon St. Mary’s Hospital, College of Medicine, The Catholic University of Korea, Bucheon, Korea; 5 Department of Internal Medicine, College of Medicine, The Chungnam National University of Korea, Daejeon, Korea; 6 National Creative Research Initiatives Center for Energy Homeostasis Regulation, Institute of Molecular Biology and Genetics and School of Biological Sciences, Seoul National University, 599 Gwanak-Ro, Gwanak-Gu, Seoul 151-742, Korea; 7 Department of Gastroenterology, University of Ulsan College of Medicine, Asan Medical Center, Seoul 138-736, Korea; 8 Convergence Medicine, University of Ulsan College of Medicine, Asan Medical Center, Seoul 138-736, Korea; 9 Division of Nephrology, Department of Internal Medicine, College of Medicine, The Catholic University of Korea, Seoul, Korea

## Abstract

Renal tubulointerstitial fibrosis (TIF) is the final pathway of various renal injuries that result in chronic kidney disease. The mammalian Hippo-Salvador signaling pathway has been implicated in the regulation of cell proliferation, cell death, tissue regeneration, and tumorigenesis. Here, we report that the Hippo-Salvador pathway plays a role in disease development in patients with TIF and in a mouse model of TIF. Mice with tubular epithelial cell (TEC)-specific deletions of *Sav1* (Salvador homolog 1) exhibited aggravated renal TIF, enhanced epithelial-mesenchymal transition-like phenotypic changes, apoptosis, and proliferation after unilateral ureteral obstruction (UUO). Moreover, Sav1 depletion in TECs increased transforming growth factor (TGF)*-*β and activated β-catenin expression after UUO, which likely accounts for the abovementioned enhanced TEC fibrotic phenotype. In addition, TAZ (transcriptional coactivator with PDZ-binding motif), a major downstream effector of the Hippo pathway, was significantly activated in *Sav1*-knockout mice *in vivo*. An *in vitro* study showed that TAZ directly regulates TGF-β and TGF-β receptor II expression. Collectively, our data indicate that the Hippo-Salvador pathway plays a role in the pathogenesis of TIF and that regulating this pathway may be a therapeutic strategy for reducing TIF.

Chronic kidney disease (CKD) is among the main causes of death and has emerged as a crucial public health issue^1^. Renal tubulointerstitial fibrosis (TIF) is the final common pathway of various renal injuries that result in CKD^2^. Renal TIF is characterized by excessive production and progressive accumulation of extracellular matrix (ECM) proteins^2^,^3^.

Transforming growth factor-β (TGF-β) signaling is known to play a crucial role in renal TIF development^4^,^5^,^6^. TGF-β initiates intracellular signaling by binding with TGF-β type II receptor (TβRII), which activates TGF-β type I receptor (TβRI), resulting in activation of downstream signaling pathways, including both SMAD-dependent and SMAD-independent pathways^5^,^6^,^7^,^8^. In most cell types, R-SMAD is phosphorylated by activated TβRI, forms a complex with SMAD4 and then translocated into the nucleus, where it regulates target genes encoding proteins involved in the fibrotic process^5^,^6^,^8^. The mechanisms that regulate TGF-β signaling are considered therapeutic targets in the treatment of renal fibrosis.

Wnt/β-catenin signaling has been shown to regulate cell proliferation and EMT (epithelial-mesenchymal transition) during embryogenesis and tumorigenesis^9^,^10^,^11^. Under Wnt-OFF conditions, a destruction complex that includes AXIN (axis inhibition protein), APC (adenomatosis polyposis coli), GSK3β (glycogen synthase kinase 3β) and CK1ɛ (casein kinase 1ɛ) phosphorylates β-catenin, which then is routed to the ubiquitin/proteasome degradation pathway. Upon Wnt stimulation, the destruction complex is inhibited, and β-catenin is released from the destruction complex and enters the nucleus, where it complexes with TCF proteins. It has been shown that Wnt/β-catenin signaling is activated in fibrotic kidney diseases and regulates renal fibrosis^12^,^13^,^14^,^15^,^16^.

The mammalian Hippo signaling pathway is an evolutionarily conserved kinase cascade that regulates cell proliferation, organ size, and tissue regeneration^17^,^18^. MST1 (mammalian Ste20-like Kinase1) and MST2, which are homologous to *Drosophila* Hippo, interact with Sav1, which is also known as WW45 (homologous to *Drosophila* Sav), and become activated by upstream signals and phosphorylate (activate) LATS1 (large tumor suppressor kinase 1) and LATS2. Activated LATS1/2 directly phosphorylates and inhibits YAP1 and TAZ (yes-associated protein; transcriptional co-activator with PDZ-binding motif), transcriptional co-activators that mainly regulate tissue development and homeostasis^19^,^20^,^21^,^22^,^23^,^24^,^25^,^26^. YAP1 can also be regulated by other upstream cues, such as G-protein-coupled receptor activation, actomyosin tension, and intracellular metabolites^27^,^28^. The Hippo signaling pathway has been shown to participate in crosstalk with other signaling pathways, such as the TGF-β and Wnt/β-catenin signaling pathways, through a variety of mechanisms^29^,^30^,^31^,^32^,^33^.

Notably, the Hippo signaling pathway plays an important role in tissue regeneration after injury. A recent study demonstrated that Yap is essential for maintaining glomerular filtration barrier integrity^34^,^35^,^36^.

In the current study, we used genetic *in vivo* and *in vitro* approaches to demonstrate the role of the Hippo signaling pathway in renal tubules in progressive TIF. We found that genetic deletion of Sav1 in TECs *in vitro* and *in vivo* substantially increased TIF severity through TGF-β and Wnt/β-catenin signaling activation.

## Results

### TEC-specific Sav1 deletions enhance TIF after UUO

**Figure 1:**
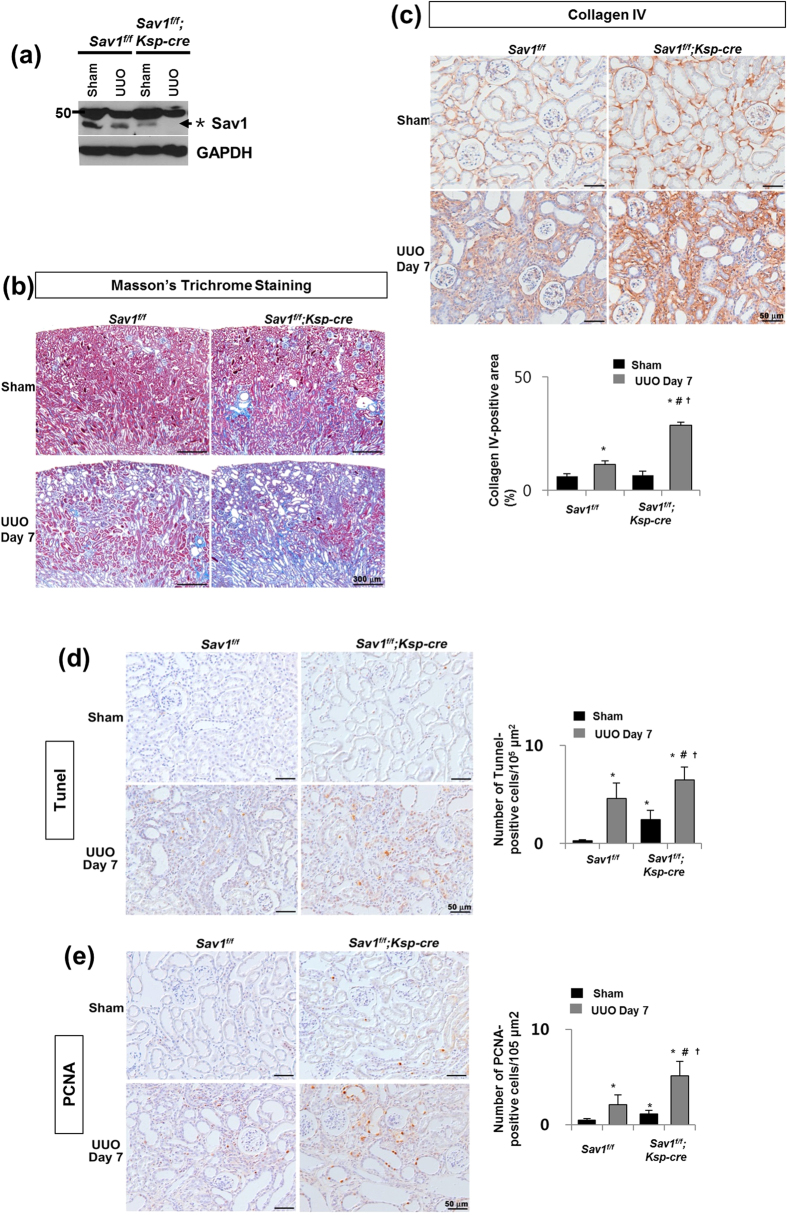
Increased TIF in Sav-depleted kidneys after UUO. (**a**) Western blot analysis of Sav1 protein expression in kidney lysates from WT and TEC-specific *Sav1*-null mice. Sav1 expression was abolished in TEC-specific *Sav1*-null mice after UUO. (*Indicates non-specific bands, and the arrow represents a verified Sav1 band). (**b,c**) TEC-specific *Sav1* deletion enhances TIF after UUO. Masson’s trichrome staining in WT and TEC-specific *Sav1*-null mice showing increased extracellular matrix deposition within the tubulointerstitium at 7 days after UUO (**b**). Immunohistochemical staining for collagen IV in WT and TEC-specific *Sav1*-null mice showing increased expression of collagen IV at 7 days after UUO (**c**). (**d,e**) TEC-specific *Sav1* deletions enhance TEC apoptosis and proliferation after UUO. Cell apoptosis and proliferation were examined by TUNEL assay (**d**) and PCNA immunostaining (**e**), respectively. TUNEL-positive cells and PCNA-positive cells were increased in injured TEC-specific *Sav1*-null mice (n = 5; ^*^P < 0.01 versus kidneys of sham-operated WT mice; ^#^*P* < 0.01 versus kidneys of sham-operated TEC-specific *Sav1*-KO mice; ^†^*P* < 0.01 versus obstructed kidneys of WT mice at 7 days after UUO). Scale bar in b = 300 μm and c, d = 50 μm.

To understand the implications of Hippo-Salvador signaling in TIF, we generated TEC-specific *Sav1*-knockout mice (*Sav1*^*fl/fl*^;*Ksp-Cre*), in which Cre expression was limited to TECs in the distal tubular segments of the kidney^37^,^38^. *Sav1*^*fl/fl*^*;Ksp-Cre* mice were born at the expected Mendelian frequencies. No overt renal histological or functional abnormalities were observed in these animals. We assessed the wild-type and floxed-alleles of the Sav1 gene using genomic PCR (Supplementary Figure 1a). On a whole-kidney homogenate level, the level of Sav1 protein expression was greatly reduced, which indicated successful generation of conditional knockout animals (Fig. 1a). The level of Sav1 protein expression was slightly decreased in wild-type kidneys after UUO, and Sav1 protein expression was also decreased in Sav1-depleted kidneys (Fig. 1a). After UUO, the progression of TIF was substantially enhanced in the kidneys of *Sav1*^*fl/fl*^*;Ksp-Cre* mice compared with those of wild-type (WT) mice. Masson’s trichrome staining demonstrated increased ECM deposition within the tubulointerstitium in WT mice at 7 days after UUO; this deposition was more severe in *Sav1*^*fl/fl*^*;Ksp-Cre* mice (Fig. 1b). Immunohistochemical staining for collagen IV confirmed the presence of exacerbated TIF in *Sav1*-null kidneys (Fig. 1c). These findings suggest that Sav1-deficient tubular epithelial cells are prone to developing more severe TIF after UUO. We investigated whether the Hippo-Salvador pathway regulates cell apoptosis and proliferation during progressive TIF by performing TUNEL (terminal deoxynucleotidyl transferase dUTP nick-end labeling) assays and immunostaining for PCNA (proliferating cell nuclear antigen), respectively. Some TUNEL-positive cells were detected in Sav1-null kidneys in sham-operation mice, but very few TUNEL-positive cells were observed in WT mice kidneys (Fig. 1d). After UUO, WT mice kidneys exhibited increased numbers of TUNEL-positive cells, an effect that was enhanced in *Sav1*^*fl/fl*^*;Ksp-Cre* mice kidneys (Fig. 1d). Similarly, immunohistochemical staining for PCNA demonstrated increased cell proliferation in Sav1-null kidneys (Fig. 1e). PCNA-positive cells were co-localized with the TEC markers NCCT (Na-Cl co-transporter) and calbindin in *Sav1*^*fl/fl*^*;Ksp-Cre* mice after UUO (Supplementary Figure S2a). Taken together, these results indicate that severe TIF induced by Sav1 deficiency is accompanied by TEC apoptosis and proliferation.

### TEC-specific Sav1 deletions increase EMT-like phenotypic changes

**Figure 2:**
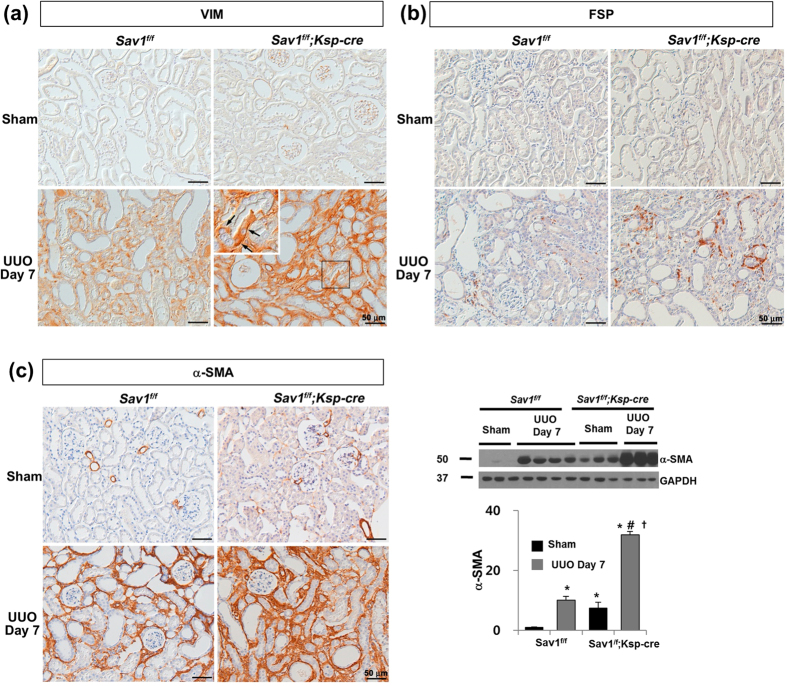
TEC-specific *Sav1* deletions promote EMT-like phenotypic changes. (**a**) Immunostaining for VIM showing a significant number of positively stained TECs in *Sav1*^*fl/fl*^*;Ksp-Cre* mice after UUO. (**b**) Immunostaining for FSP-1. (**c**) Immunostaining and immunoblotting for α-SMA (n = 5; ^*^P < 0.01 versus kidneys of sham-operated WT mice; ^#^*P* < 0.01 versus kidneys of sham-operated TEC-specific *Sav1*-null mice; ^†^*P* < 0.01 versus obstructed kidneys of WT mice at 7 days after UUO). Scale bar in a, b, and c = 50 μm.

To determine whether EMT-like phenotypic changes contribute to TIF development in Sav1-null kidneys after UUO, we observed EMT marker expression. The expression of α-SMA, a marker of myofibroblasts^3^, was markedly upregulated in the obstructed kidneys of *Sav1*^*fl/fl*^*;Ksp-Cre* mice compared with the obstructed kidneys of WT mice (Fig. 2c and Supplementary Figure S2b). Immunohistochemical staining for vimentin (VIM), a cytoskeleton protein and a specific marker of mesenchymal cells, demonstrated significant numbers of VIM-positive TECs in obstructed Sav1-null kidneys after UUO (Fig. 2a). VIM transcripts were substantially increased in *Sav1*^*fl/fl*^*;Ksp-Cre* mice compared with control mice after UUO (Supplementary Figure S2b). Additionally, the numbers of interstitial cells expressing fibroblast-specific protein-1 (FSP-1) were also increased in *Sav1*^*fl/fl*^*;Ksp-Cre* mice compared with control mice after UUO (Fig. 2b). The mRNA expression levels of CDH1 (E-cadherin), an epithelial cell marker^39^, were decreased in Sav1-null mice compared with controls after UUO (Supplementary Figure S2b). These data suggest that Hippo-Salvador pathway dysfunction induces tubular EMT-like phenotypic changes after UUO.

**Figure 3:**
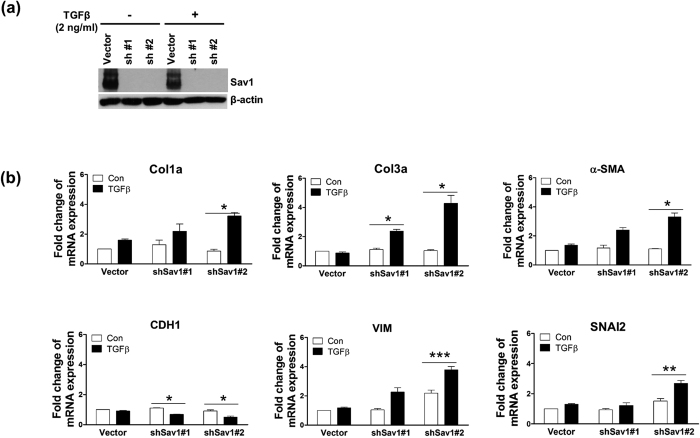
Sav1 depletion alters TGFβ-induced EMT marker expression. (**a**) Western blot analysis of Sav1 expression in HK2 cells transduced with shRNA lentiviruses against Sav1 or control vectors and incubated with TGFβ for 12 hours. (**b**) qRT-PCR analysis of EMT markers from cells confirmed in (**a**). Col1a, Col3a, α-SMA, VIM, and SNAI2 expression was enhanced in Sav1-depleted HK2 cells exposed to TGF-β. CDH1 expression was decreased in the same cells. Error bars represent SEMs. ^*^P < 0.05; ^**^P < 0.01; ^***^P < 0.005 by paired t-test (one-tailed).

To determine whether Sav1 depletion induces TGF-β-induced EMT-like phenotypic changes *in vitro*, we knocked down Sav1 in HK2 cells via lentiviral delivery of small hairpin (interfering) RNA (shRNA; #1 and #2) molecules against Sav1 and then treated the cells with TGF-β1 for 12 hours (Fig. 3a). Treatment of Sav 1-depleted HK2 cells with TGF-β1 caused marked increases in Col1a (alpha-1 type I collagen), Col3a, α-SMA, VIM, and SNAI2 (snail family zinc finger 2) mRNA expression (Fig. 3b). By contrast, exposure to TGF-β1 reduced CDH1 mRNA levels by ~50% in Sav11-depleted HK2 cells compared with untreated Sav1 cells. These results suggest that Sav1 deficiency results in tubular EMT-like phenotypic changes after TGF-β1 treatment in an *in vitro* cell culture system.

### YAP1/TAZ activation in the setting of Sav1 deficiency induces TGF-β2 and TβRII expression

**Figure 4:**
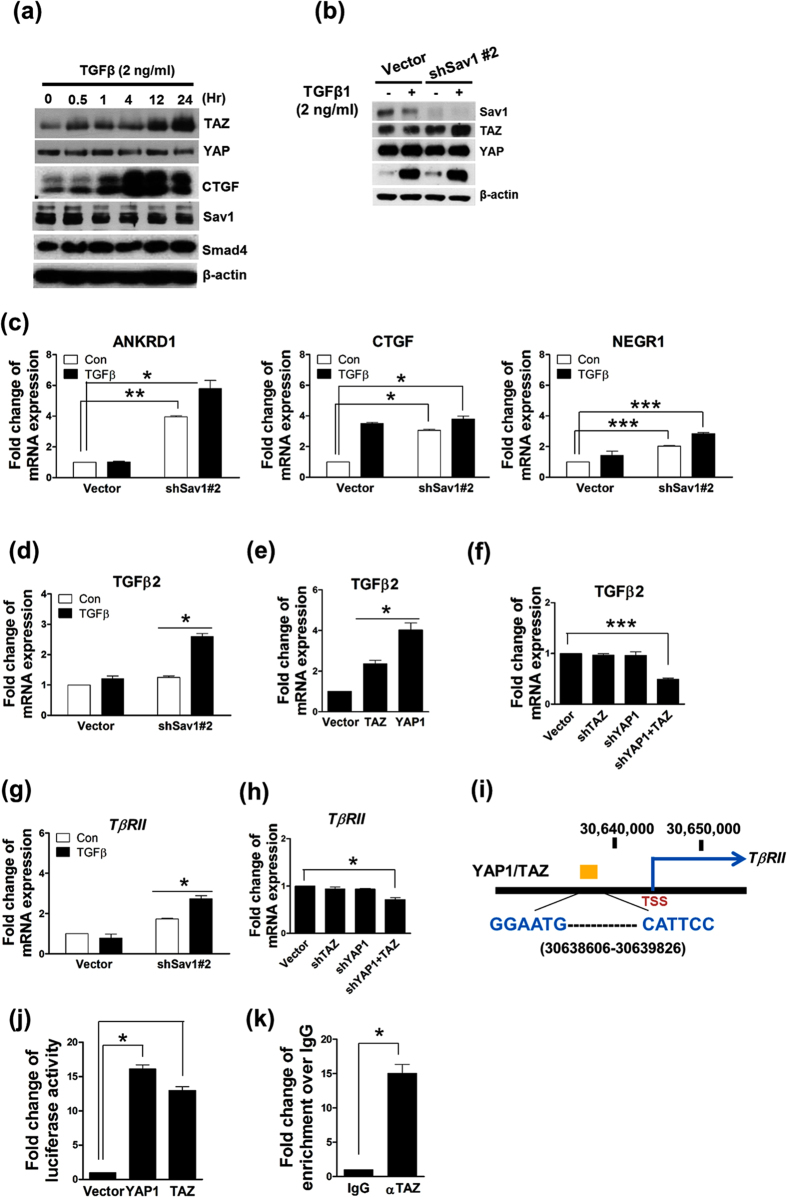
YAP1/TAZ induces TGF-β2 expression and directly upregulates *TGF-βRII* expression *in vitro*. (**a**) Western blot analysis of YAP1, TAZ, Sav1, CTGF, and SMAD4 expression in HK2 cells incubated with TGF-β1 after serum starvation for 18 h. (**b**) Western blot analysis of the indicated proteins in HK2 cells expressing control vectors or shRNA lentiviral vectors against Sav1. (**c**) qRT-PCR analysis of ANKRD1, CTGF, and NEGR1 expression in HK2 cells…*P* = 0.0073, 0.001236, 0.00061 (comparison between the 1st bar and 3rd bar) and 0.03576, 0.02104, 0.00083 (comparison between the 1st bar and 4th bar) for ANKRD1, CTGF and NEGR1, respectively. (**d–f**) Upregulation of TGF-β2 expression by YAP1/TAZ.. qRT-PCR analysis of TGF-β2 mRNA expression in HEK293 cells transduced with (**d**) Sav1-depleting shRNA vectors or control vectors and incubated with (dark gray bars) or without (light gray bars) TGF-β1 for 12 hours. *P* = 0.03522 (comparison between the 3rd bar and 4th bar). (**e**) TAZ or YAP1 expression plasmids and (**f**) TAZ-, YAP1- or TAZ/YAP1-depleting shRNA vectors. *P* = 0.00093 (comparison between the 1st bar and 4th bar). (**g**) The increase in *TβRII* mRNA expression elicited by TGF-β1 treatment in the Sav1 deficiency. qRT-PCR analysis of *TβRII* mRNA expression in Sav1 depleted-HK2 and control cells treated with or without TGF-β1. *P* = 0.03204 (comparison between the 3rd bar and 4th bar). (**h**) Downregulation of *TβRII* mRNA expression by deletion of both YAP1 and TAZ in HEK293 cells.. *P* = 0.0389 (comparison between the 1st bar and 4th bar). (**i**) Schematic of part of the *TβRII* genomic region containing a YAP1/TAZ-enriched site showing TEAD binding sequences (GGAATG/CATTCC) located between 30638606 and 30639826 in chromosome 3, a YAP1 or TAZ-enriched region (yellow box), and the *TβRII* transcription start site (TSS). (**j**) Luciferase activity assays of the *TβRII* genomic upstream region shown in (**i**). 293T cells transfected with an pGL3 vector containing a genomic fragment including the YAP1/TAZ-enriched region were co-transfected with YAP1 or TAZ expression plasmids or control vectors. (**k**) ChIP assay for TAZ-enriched regions. immunoprecipitated DNA with an anti-Flag antibody was amplified with specific primers covering potential TEAD binding sequences. All error bars represent SEMs. ^*^P < 0.05; ^**^P < 0.01; ^***^P < 0.005 by paired t-test (one-tailed).

Our results suggest that Sav1 deficiency induces TGF-β-induced EMT-like phenotypic changes. Furthermore, recent studies have shown that YAP1 and TAZ cooperate with Smad proteins to promote TGF-β signaling. For example, a study reported that YAP1/TAZ act as mechanoregulators^40^ and contribute to the development of renal fibrosis via promotion of TGF-β signaling^41^. We first tested whether TAZ or YAP1, which are major targets of the Hippo pathway, plays a role in TGF-β signaling in renal TIF. We examined time courses of the changes in TAZ and YAP1 protein expression during incubation with TGF-β *in vitro*. TGF-β1 treatment increased TAZ protein expression in a time-dependent manner but did not affect YAP1 protein expression (Fig. 4a). CTGF is a well-characterized YAP/TAZ target gene. TGF-β1 induced CTGF expression, which means that YAP and TAZ were activated by TGF-β1 (Fig. 4a). We also examined the levels of proteins involved in the Hippo pathway in Sav1-depeleted cells. As shown in Fig. 4b, TGF-β1 treatment clearly activated SMAD3 in HK2 cells. Furthermore, TAZ protein levels increased in Sav1-depleted cells in response to TGF-β treatment (Fig. 4b). However, YAP1 protein levels were unchanged between the control and Sav1-depeleted cells, irrespective of the presence of TGF-β1 (Fig. 4b).

To confirm that increases in TAZ protein expression affect Hippo pathway target gene expression, we examined the effects of Sav1 deletion on the expression of the following YAP1/TAZ target genes: *ANKRD1* (ankyrin repeat domain 1), *NEGR1* (neuronal growth regulator 1), and *CTGF* (connective tissue growth factor). The expression levels of ANKRD1, NEGR1, and CTGF mRNA were elevated in Sav1-deficient cells but were not increased by TGFβ1 treatment (Fig. 4c). These data indicate that increases in TAZ protein expression induced by Sav1 depletion lead to enhanced TAZ-mediated transcriptional activation of *ANKRD1, NEGR1,* and *CTGF*; however, Sav1 does not appear to significantly alter the levels of YAP1 protein expression. It has been reported that YAP1 positively regulates TGF-β2 expression^42^.

We therefore tested whether increased TAZ activation in Sav1-deficient cells is accompanied by enhanced TGF-β2 expression. As shown Fig. 4d, TGF-β2 mRNA levels were increased in Sav1-depleted cells that were preincubated with TGF-β1 compared with control cells. We also found that YAP1 and TAZ overexpression stimulated TGF-β2 expression (Fig. 4e). Furthermore, knockdown of YAP1 or TAZ individually did not significantly reduce TGF-β2 expression; however, depletion of both YAP1 and TAZ significantly reduced TGF-β2 expression (Fig. 4f). These data suggest that YAP1 activation or increased TAZ protein expression in the setting of Sav1 depletion upregulates TGF-β2 expression, thereby contributing to TGF-β pathway activation; however, YAP1 protein expression was not altered in Sav1-depleted cells.

We previously noted that YAP1 protein was enriched in the genomic region of TβRII (TGF-β receptor II) in human MCF10A cells (unpublished data) and was potentially bound to the genomic region of *TβRII* in embryonic stem cells^43^. TAZ, a paralog of YAP1, also appeared to be enriched in the same region. These observations prompted us to investigate whether *TβRII* expression is directly regulated by YAP1/TAZ. *TβRII* mRNA expression was increased to greater extent in Sav1-depleted cells than in control cells and was increased by TGF-β treatment (Fig. 4g). Notably, knockdown of both YAP1 and TAZ reduced *TβRII* expression (Fig. 4h). To investigate the direct regulation of *TβRII* expression by YAP1/TAZ, we searched the *TβRII* genomic region based on an analysis of our unpublished YAP1-Chip sequencing profile data, which we integrated with TEAD4-Chip sequencing data provided by the UCSC browser. We identified one conserved genomic region upstream of the transcription start site of *TβRII* that may be bound by YAP1/TAZ and TEAD4 (Fig. 4i, yellow box). To verify this genomic region, we tested whether this region-driven luciferase reporter is activated by YAP or TAZ overexpression. We found that YAP1 and TAZ expression dramatically enhanced the luciferase activity of the promoter-reporter construct (Fig. 4j). We also confirmed that TAZ bound directly to the *TβRII* genome by performing ChIP (chromatin immunoprecipitation) assays using an anti-FLAG antibody in HEK293 cells expressing Flag-tagged TAZ. These assays showed that TAZ was highly enriched in the conserved region upstream from the *TβRII* transcription start site (Fig. 4k). Taken together, these results suggest that activation of TGF-β signaling by Sav1 deficiency is associated with increases in TGF-β2 and *TβRII* expression mediated by YAP1/TAZ activation.

### TAZ and YAP1 are implicated in renal TIF *in vivo*

**Figure 5:**
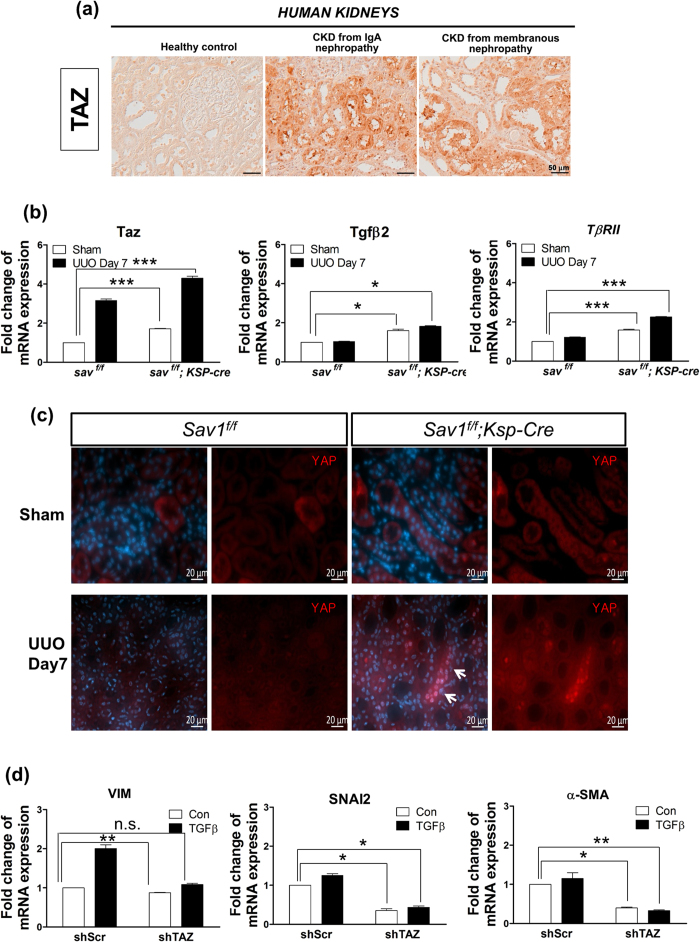
TAZ protein levels are increased in the kidneys of patients with CKD. (**a**) Immunohistochemical staining for TAZ expression in the kidneys of a healthy control individual and of patients with biopsy-documented IgA nephropathy and membranous nephropathy. (**b**) Relative TAZ and TGF-β2 expression and *TβRII* transcript levels were measured in the kidneys of WT and TEC-specific *Sav1*-null mice after UUO using qRT-PCR. Sav1-null mice kidneys exhibited increased TAZ, TGF-βII, and *TβRII* mRNA expression. Error bars represent SEMs. ^*^P < 0.05; ^**^P < 0.01; ^***^P < 0.005 by paired t-test (one-tailed). *P* = 0.00043, 0.03991, 0.00254 (comparison between the 1st bar and 3rd bar) and 0.00048, 0.01366, 0.00024 (comparison between the 1st bar and 4th bar) for ANKRD1, CTGF and NEGR1, respectively. (**c**) Sav1 deficiency after UUO resulted in nuclear accumulation of YAP1. Immunofluorescence staining for YAP1 (Red) with anti-YAP1 antibodies in control and TEC-specific *Sav1*-null mice. YAP1 was strongly localized in the nuclei of SAV1-nul kidneys after UUO. (**d**) qRT-PCR analysis of the EMT markers VIM, SNAI2, and α-SMA in control and TAZ-knockdown HK2 cells during incubation with TGF-β1. TAZ knockdown reduced TGF-β1-induced EMT marker expression. Error bars represent SEMs. n.s., not significant; ^*^P < 0.05; ^**^P < 0.01 by paired t-test (one-tailed). Scale bar in a = 50 μm and c = 20 μm.

Given that TGF-β1 treatment increased TAZ protein levels, but not YAP1 protein levels (Fig. 4a,b), and that Sav1 depletion activated YAP1/TAZ target gene transcription (Fig. 4c), we examined TAZ mRNA expression in kidney tissues and assessed the functional role of TAZ in renal fibrosis. TAZ mRNA expression was increased in Sav1-null kidneys after UUO (Fig. 5b). Consistent with this finding, TAZ was present at high levels in the kidneys of CKD patients (Fig. 5a). We also tested whether TGF-βII and *TβRII* transcript levels are altered in Sav1-null mice exhibiting high TAZ expression. Sav1 depletion resulted in high TGF-βII and *TβRII* mRNA expression after UUO (Fig. 5b). As TGF-βII and *TβRII* gene expression is upregulated in Sav1-null kidneys, we investigated the localization of Smad4 as a marker of TGF-β signaling activation. Smad4 exhibited significant accumulation in the nuclei of UUO-treated Sav1-null kidneys (Supplementary Figure 3). Previous results demonstrated that knockdown of both YAP1 and TAZ downregulated TGF-β2 and TβRII mRNA expression, even though YAP1 total protein expression was not affected in Sav1-depleted HK2 cells (Fig. 4f,h), indicating that YAP1 activation is required for TGFβ-signaling. We therefore surmised that the cellular localization of YAP1 is altered, as opposed to its protein levels. To determine whether YAP1 cellular localization is altered in Sav1-depleted kidneys after UUO, we observed YAP1 cellular localization in the TECs of control kidneys and Sav1-null kidneys after UUO (Fig. 5c). Irrespective of UUO, the basal levels of YAP1 protein expression were low, and YAP1 cellular localization was not altered in control kidneys. However, YAP1 accumulated in the nucleus in Sav1-null kidneys (Fig. 5c). suggesting that Sav

1 deficiency leads to nuclear accumulation of YAP1, which alters gene expression. We next determined whether TAZ plays a role in renal TIF via regulation of EMT-like phenotypic changes *in vitro*. Interestingly, TGF-β1-induced increases in VIM expression were remarkably reduced in the absence of TAZ (Fig. 5d). Similarly, knockdown of TAZ led to strong reductions in SNAI2 and α-SMA expression, which persisted even during stimulation with TGF-β1 (Fig. 5d). Taken together, these results suggest that activation of TAZ in Sav1 deficiency plays a role in renal TIF by strongly triggering EMT-like phenotypic changes.

### Sav1 deficiency increases β-catenin activation *in vitro* and *in vivo*

**Figure 6:**
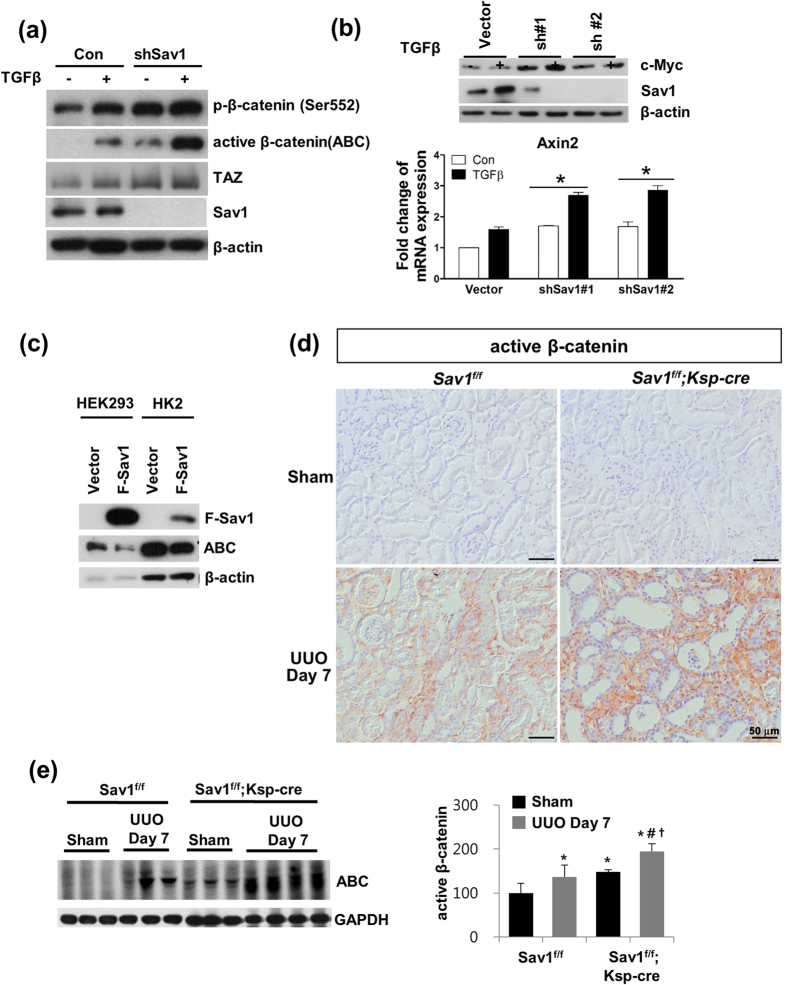
A Sav1 deficiency activates Wnt/β-catenin signaling. (**a**) Western blot analysis of Ser552-phosphorylated β-catenin, unphosphorylated (active) β-catenin (ABC), TAZ, and Sav1 in control and Sav1-depleted HK2 cells with or without TGF-β1 treatment. Active β-catenin expression was enhanced by Sav1 deficiency and was increased by TGF-β1 treatment. (**b**) Expression of Wnt target genes c-MYC and AXIN2 in Sav1-deficient (shRNA #1 and #2) HK2 cells. *Top:* Western blot analysis of c-MYC expression. *Bottom:* qRT-PCR analysis of AXIN2 expression. c-MYC and AXIN2 expression was increased in TGF-β1-treated, Sav1-deficient HK2 cells. Error bars represent SEMs. ^*^P < 0.05 by paired t-test (one-tailed). (**c**) Western blot analysis of active β-catenin expression in Sav1-overexpressing cells. HEK293 and HK2 cells were transfected with a Flag-Sav1 expression construct or vector control, and active β-catenin expression was detected. Sav1 overexpression reduced the level of active β-catenin expression. (**d**) Immunohistochemical staining for active β-catenin in the kidneys of WT and *Sav1*-null mice after UUO. *Sav1*-null mice kidneys stained strongly for active β-catenin after UUO. (**e**) Western blot analysis of active β-catenin expression *in vivo*. Active β-catenin expression was highly increased in the kidney tissues of TEC-specific, *Sav1*-null mice after UUO. Scale bar in d = 50 μm.

Aberrant activation of Wnt/β-catenin signaling is a common pathologic finding in renal fibrosis. Therefore, we investigated whether Wnt/β-catenin signaling is activated in Sav1-depleted cells. As shown in Fig. 6a, Ser552-phosphorylated β-catenin and active β-catenin levels were increased in Sav1-depleted cells compared with control cells and were increased to a greater extent in Sav1-depleted cells than in control cells by TGF-β1 treatment (Fig. 6a). A functional consequence of increased β-catenin activity in Sav1-deficient cells (shSav #1-treated or #2-treated) was increased expression of the Wnt/β-catenin target genes c-MYC and AXIN2 at the mRNA and protein level; these increases, especially that of AXIN2, were enhanced by TGF-β treatment (Fig. 6b). Conversely, Sav1 overexpression reduced active β-catenin protein levels in HEK293 and HK2 cells, suggesting that Sav1 protein negatively regulates Wnt/β-catenin signaling (Fig. 6c). Consistent with these *in vitro* data, we observed that active β-catenin expression was increased after UUO in the obstructed kidneys of WT mice compared with the kidneys of sham-operated mice and was substantially enhanced in the obstructed kidneys of *Sav1*^*fl/fl*^*;Ksp-Cre* mice (Fig. 6d,e). These results suggest that Sav1 deficiency is associated with increased β-catenin activity in renal fibrosis.

## Discussion

In this study, we demonstrated that the Hippo signaling pathway plays a pivotal role in the development of renal fibrosis. We focused on Sav1 deletion and subsequent TAZ activation in the Hippo signaling pathway as regulators of fibrosis in CKD because the expression of TAZ protein was markedly increased in mouse Sav1-depleted kidneys after UUO and in patients with TIF/CKD. In addition, we found that Sav1 depletion leads to YAP1 nuclear accumulation after UUO. UUO-induced TIF in renal TECs lacking Sav1 is aggravated by increased TAZ protein expression and YAP1 nuclear localization, resulting in TGF-β and *TβRII* expression upregulation and aberrant activation of Wnt/β-catenin signaling activation.

Several studies have reported the occurrence of crosstalk between the Hippo pathway and TGF-β signaling. For example, TGF-β stimulates the binding of TAZ to activated SMAD complexes in the nucleus, thereby enhancing responsiveness to TGF-β signaling^30^,^31^. However, we did not observe strong interactions between TAZ and SMADs in Sav1-deleted cells, even in the presence of TGF-β. Accordingly, other proteins may be involved in the crosstalk between the Hippo pathway and TGF-β signaling. In fact, YAP1 has been shown to regulate TGF-β^43^,^44^. We also confirmed that YAP1 and TAZ overexpression increased TGF-β2 expression *in vitro* (Fig. 4e) and that YAP1 and TAZ knockdown reduced TGF-β2 expression. Sav1 deficiency elevated TGF-β2 and TGF-βRII mRNA expression *in vivo* and *in vitro* by increasing TAZ protein levels and YAP1 activity (Fig. 4d,g and Fig. 5b). Moreover, we demonstrated that TAZ directly induces *TβRII* mRNA (Fig. 4k). Conversely, TAZ is known to be transcriptionally induced by TGF-β1^30^. Consistent with this finding, TAZ was upregulated over time by TGF-β1 *in vitro* (Fig. 4a), and its mRNA expression remained at a high level *in vivo* after UUO (Fig. 5b). However, neither TGF-β1 treatment nor Sav1 depletion affected cellular YAP1 protein levels. Interestingly, subcellular YAP1 localization was strongly maintained in renal TECs lacking Sav1 after UUO. Given these observations, we assumed that the UUO procedure itself stimulates TGF-β1 secretion, resulting in increased TAZ activation and YAP1 nuclear accumulation in the setting of Sav1 depletion, as well as subsequent TGF-β signaling activation by TGF-β2 and *TβRII* induction through TAZ activation. In line with these results, TAZ depletion prevented induction of the expression of the EMT markers VIM, SNAI2, and α-SMA in response to TGF-β treatment *in vitro* (Fig. 5d). Based on these results, we propose that YAP1/TAZ activation after UUO in TEC-specific *Sav1*-null mice promotes TGF-β-induced EMT-like phenotypic changes that contribute to renal TIF. In addition, CTGF, which is encoded by a known direct target gene of TGF-β and YAP1/TAZ, induces sustained fibrosis along with TGF-β^37^,^45^. Our observations indicate that CTGF mRNA expression was enhanced by YAP1/TAZ activation, which may exacerbate renal fibrosis.

Wnt/β-catenin signaling activation is a common feature in a variety of CKDs. Notably, β-catenin plays a critical role in renal fibrosis. The current study also showed that Sav1 depletion resulted in increased active β-catenin expression and revealed that TGF-β treatment and UUO enhanced Wnt/β-catenin signaling activity. Unfortunately, the mechanism linking the loss of Sav1 to the increase in active β-catenin expression remains unclear. It is reasonable to assume that high levels of active β-catenin protein expression are not directly induced by Sav1 depletion but instead may be a secondary effect of intrinsic TGF-β signaling activation by Sav1 deletion. This idea is supported by the previous finding that TGF-β signaling itself upregulates Wnt/β-catenin signaling through suppression of Dkk1 (Dickkopf WNT signaling pathway inhibitor 1) or Klotho mRNA expression^46^,^47^.

**Figure 7:**
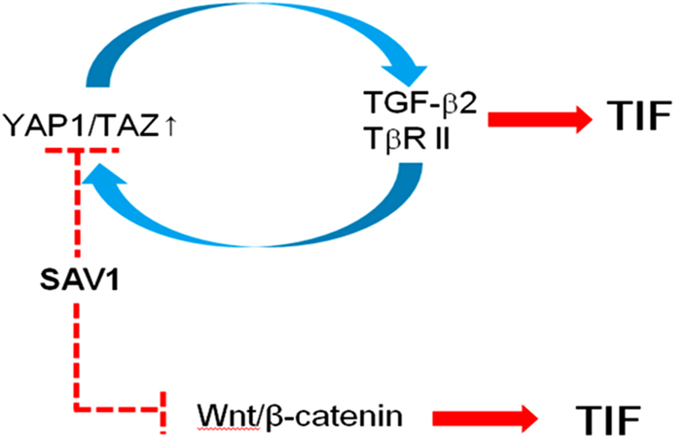
Schematic representation of the proposed role of the Hippo-Salvador signaling pathway in tubular epithelial cells in renal tubulointerstitial fibrosis. YAP1/TAZ activation in Sav1-depleted cells induces TGF-β and *TβRII* mRNA expression, thereby synergistically enhancing responsiveness to TGF-β. This mechanism promotes TGF-β-induced EMT-like phenotypic changes and contributes to progressive renal TIF. Sav1 depletion also enhances Wnt/β-catenin activation, which exacerbates TIF.

In summary, our data show for the first time that the Hippo-Salvador pathway regulates renal TIF through TGF-β and Wnt/β-catenin signaling (Fig. 7). Our results indicate that the Hippo-Salvador pathway is a new mechanism underlying the pathogenesis of TIF development and that regulation of Hippo signaling may represent a therapeutic strategy for mitigating TIF.

## Materials and Methods

### Human kidney specimens

Human kidney samples were obtained from nine archived kidney biopsy specimens from patients with chronic renal TIF (three IgA nephropathy and two membranous nephropathy) and from normal kidney tissue specimens obtained via nephrectomy at the Bucheon St. Mary’s Hospital Department of Pathology. The study was approved by the Medical Ethics Committee of Bucheon St. Mary’s Hospital.

### Animals

Floxed *Sav1* mice (*Sav1*^fl/fl^; previously designated *WW45*^fl/fl^) were generated as described previously^27^. To generate mice with a *Sav1* deletion specifically in the tubules, we crossed *Sav1*^*fl/fl*^ mice with transgenic *Ksp-Cre* mice, which were purchased from the Jackson Laboratory (Bar Harbor, ME, USA). The genotypes of the offspring were determined by polymerase chain reaction (PCR) analysis using genomic DNA obtained from the tails of mice and transgene-specific primers. All mouse lines were bred onto a C57BL/6 background. Only male mice (20–25 g, 8 weeks old) were used in this study. UUO was performed using an established procedure. All animal experiments were approved by the Ethics Committee of Bucheon St. Mary’s Hospital.

### Histological analysis

Formalin-fixed, paraffin-embedded kidney sections were stained with hematoxylin-eosin (HE), periodic acid Schiff (PAS), and Masson’s trichrome using standard protocols.

### Immunohistochemistry

Immunohistochemical staining was performed according to a previously established protocol^28^. Primary antibodies against ACTA2 (Sigma-Aldrich, St. Louis, MO, USA), VIM (Santa Cruz Biotechnology, California, USA), FSP-1 (Thermo Scientific, Fremont, CA, USA), PCNA (Dako, Glostrup, Denmark), activated β-catenin (Sigma-Aldrich), and TAZ (Santa Cruz Biotechnology, Santa Cruz, CA, USA) were used. TUNEL staining was performed using a commercial kit (Millipore, Billerica, MA, USA), in accordance with the manufacturer’s instructions.

### Immunofluorescence staining and confocal microscopy

Immunofluorescence procedures were performed as previously described^28^. Cell proliferation was detected using a primary polyclonal antibody against PCNA (Abcam, ab2426). Immunolabeled proteins were visualized using Alexa 647-conjugated donkey anti-rabbit (Invitrogen, Grand Island, NY, USA), Cy3-conjugated donkey anti-chicken (Jackson ImmunoResearch laboratories Inc., West Grove, PA, USA), and FITC-conjugated donkey anti-goat antibodies (Jackson ImmunoResearch Lab), as appropriate. For YAP1 immunofluorescence staining, anti-YAP1 antibody was used (Cell Signaling, cat 4912 and Anti-rabbit IgG (H + L), F(ab’)2 Fragment (Alexa Fluor^®^ 594 Conjugate) cat 8889). Tissues were mounted in Vectashield mounting medium (Vector Laboratories, CA, USA). Images were acquired using a Zeiss LSM 510 confocal microscope (Carl Zeiss, Germany) and LSM 510 version 2.02 software.

### Western blot analysis

Kidney tissues were lysed on ice with radioimmunoprecipitation assay (RIPA) buffer (1% NP-40, 0.1% SDS, 100 μg/ml PMSF, 1% protease inhibitor cocktail, 1% phosphatase I and II inhibitor cocktails in phosphate-buffered saline [PBS]). The supernatants were collected after centrifugation, and protein expression was analyzed via Western blotting, as described previously^28^. The following primary antibodies were used: anti-Sav1 (rabbit polyclonal, Ctr#1)^29^, anti-TAZ (Santa Cruz Biotechnology), anti-fibronectin (Dako), anti-collagen I (Southern Biotech, Birmingham, AL, USA), anti-ACTA2 (Sigma-Aldrich), anti-TGF-β (R&D Systems, Minneapolis, Minnesota, USA), anti-SMAD2 (Invitrogen Corporation, Camarillo, CA, USA), anti-SMAD3 (Cell Signaling Technology, Beverly, MA, USA), anti-SMAD4 (Santa Cruz Biotechnology), anti-activated β-catenin (Sigma-Aldrich), and anti-GAPDH (Santa Cruz Biotechnology).

For Western blot analysis of cultured cells, cells were washed with PBS and harvested with 1 mM EDTA in PBS. Cells were then lysed with lysis buffer containing 0.5% Triton X-100 and cleared by centrifugation. Protein concentrations were measured using the Bradford method. Proteins in cell lysates were resolved by sodium dodecyl sulfate-polyacrylamide gel electrophoresis (SDS-PAGE) and transferred to a PVDF (polyvinylidene difluoride) membrane. The membrane was blocked in 5% non-fat dry milk for 1 hour and incubated with primary antibodies diluted in 5% bovine serum albumin (BSA). The membrane was then washed, incubated with horseradish peroxidase-linked secondary antibodies (Jackson Laboratory) diluted 1:5000 in 5% non-fat dry milk, and developed using an enhanced chemiluminescence kit (Amersham, PA, USA). The indicated commercial primary antibodies against the following proteins were used for Western blotting: TAZ (Cell Signaling, #4883), SMAD3 (Cell Signaling, #9523), phospho-SMAD3 (S423/425; Cell Signaling, #9520), SMAD4 (Santa Cruz Biotechnology, sc-7966), active β-catenin (dephospho S37 or T41; Millipore, 05–665), phospho-β-catenin (S552; Cell Signaling, #9566), total β-catenin (Santa Cruz Biotechnology, sc-7199), YAP (Cell Signaling, #4912), phospho-YAP (S127; Cell Signaling, #4911), LATS2 (Cell Signaling, #5888), LATS1 (Bethyl Laboratories, TX, USA, A300–477A), β-actin (Sigma, A5316), and Flag (Wako, 012–22384). The rabbit anti-Sav1 antibodies have been previously described (Lee *et al*., 2008).

### Cell culture and viral infection

293T cells and HEK293 cells were cultured in Dulbecco’s modified Eagle medium (DMEM) supplemented with 10% fetal bovine serum (FBS). HK-2 cells were cultured in DMEM/F12 containing 10% FBS. For TGF-β1 treatment, cells were cultured in serum-free medium for 18 h and then treated with or without TFG-β1 (2 ng/ml) for 12 h. Flag-YAP1, Flag-TAZ, and Flag-Sav1 were cloned into a retroviral pMSCV plasmid. shYAP1 and shTAZ were cloned into Super Retro plasmids. 293T cells were transfected with retroviralvectors (5:5:2 ratio of retroviral vector:Gag-pol:VSVG) using the calcium precipitation method. Viral supernatants were collected 1 day after transfection. Three days after transfection, viral supernatants were cleared by centrifugation at 3000 rpm for 20 minutes and filtered using a 0.45-μm filter (Millipore). Target cells were infected with the resulting viruses using 6 μg/ml polybrene (Sigma, 107689) and were selected 2 days later by culturing in the presence of 3–6 μg/ml puromycin. shSav1 was cloned into the pLKO.1-puro lentiviral vector. 293T cells were transfected with lentiviruses (6:4.5:4.5:3 ratio of lentiviral vector:P1:P2:VSVG) using the calcium phosphatase method. Virus collection, infection and selection were performed using the same methods as those used for the retroviruses.

### Analysis of gene expression by quantitative real-time PCR

Total RNA was prepared using the TRIzol reagent (Invitrogen), as directed by the manufacturer. Quantitative real-time PCR analyses were carried out as previously described^48^ using primers described in Supplemental Information.

### Luciferase assays

Genomic fragments of TβRII, which were generated by PCR, were subcloned into an FGF4 minimal promoter-linked pGL3 vector expressing Firefly luciferase and co-transfected into cells with YAP1 or TAZ expression plasmids or control vectors, as well as a Renilla luciferase expression plasmid, using a Dual-Luciferase Reporter Assay (Promega), as directed by the manufacturer. Luciferase activity was measured and normalized to Renilla activity to control for transfection efficiency.

### ChIP-Seq and data analysis

ChIP-Seq procedures and data analysis were performed as previously described^46^. The sequences of the primers are described in Supplemental Figure S3.

### Statistical analyses

Results are presented as means ± SDs. A t-test was used for comparisons between two groups. Differences among two or more groups were compared using one-way analysis of variance (ANOVA). *P*-values less than 0.05 were considered significant.

### Study approval

Human kidney samples were obtained from patients with chronic renal TIF and from normal kidney tissue specimens obtained via nephrectomy at the Bucheon St. Mary’s Hospital Department of Pathology. The study was approved by the Medical Ethics Committee of Bucheon St. Mary’s Hospital (HC14SISI0048). All human data and samples used in this study were anonymized. All identifiers of human data or samples were irreversibly stripped via an arbitrary alphanumeric code, making it impossible for anyone to link the samples to their sources. Therefore, no informed consent was obtained from the research participants. All experimental procedures were performed according to animal care and ethics legislation, and the study was approved by the Animal Care Committee of Bucheon Saint Mary’s Hospital.

## Additional Information

**How to cite this article**: Seo, E. *et al*. The Hippo-Salvador signaling pathway regulates renal tubulointerstitial fibrosis. *Sci. Rep.* **6**, 31931; doi: 10.1038/srep31931 (2016).

## Supplementary Material

Supplementary Information
