# Supplementary material for: The Hippo-Salvador signaling pathway regulates renal tubulointerstitial fibrosis
Source: Sci Rep. 2016 Aug 23;6:31931. doi: 10.1038/srep31931 (PMC4994041; doi:10.1038/srep31931)
Supplement: Supplementary Information [file srep31931-s1.pdf]

The Hippo-Salvador signaling pathway regulates renal tubulointerstitial fibrosis

Eunjeong Seo, Wan-Young Kim, Jeongmi Hur, Hanbyul Kim, Sun Ah Nam, Arum Choi, Yu-Mi Kim, Sang Hee Park, Chaeuk Chung, Jin Kim, Soohong Min, Seung-Jae Myung, Dae-Sik Lim and Yong Kyun Kim

Supplementary figures 1

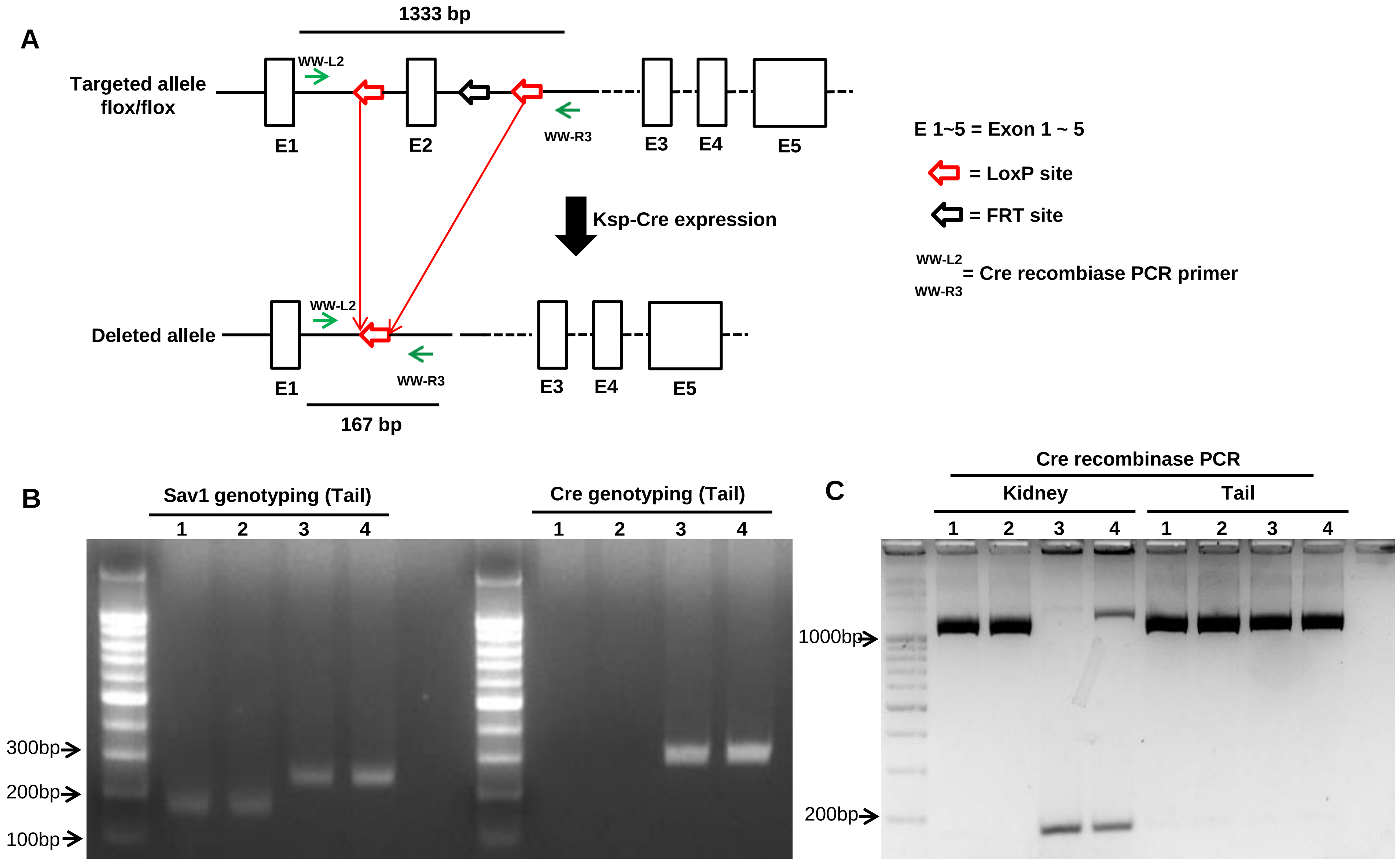

1. Sav1 +/+   3. Sav1 $^{F/F}$; Ksp-cre

2. Sav1 +/+   4. Sav1 $^{F/F}$; Ksp-cre

1. Sav1 +/+   3. Sav1 $^{F/F}$; Ksp-cre

2. Sav1 +/+   4. Sav1 $^{F/F}$; Ksp-cre

Supplementary figures 2

a

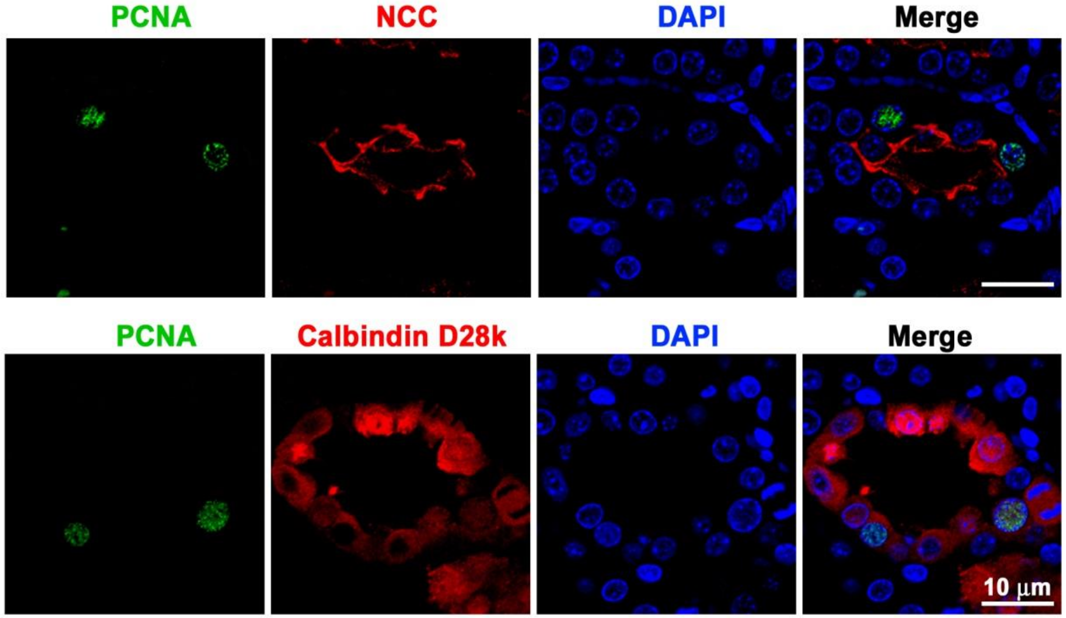

b

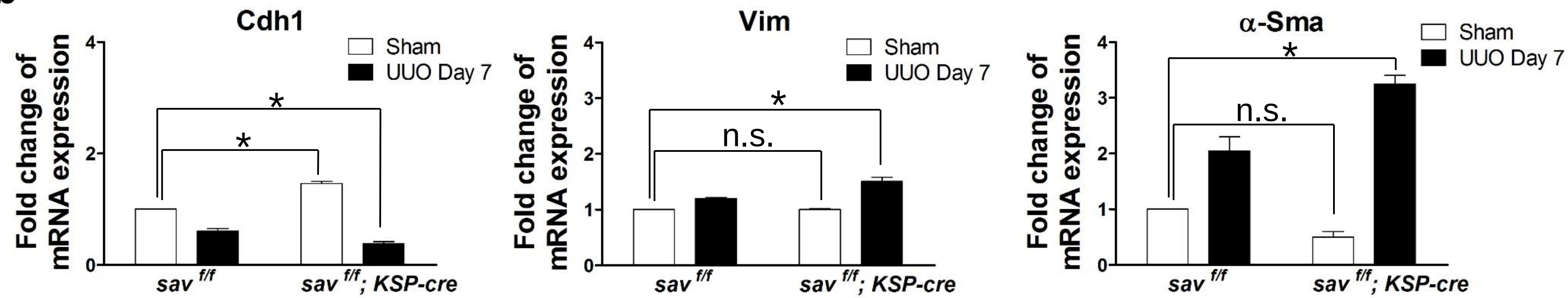

Supplementary figures 3

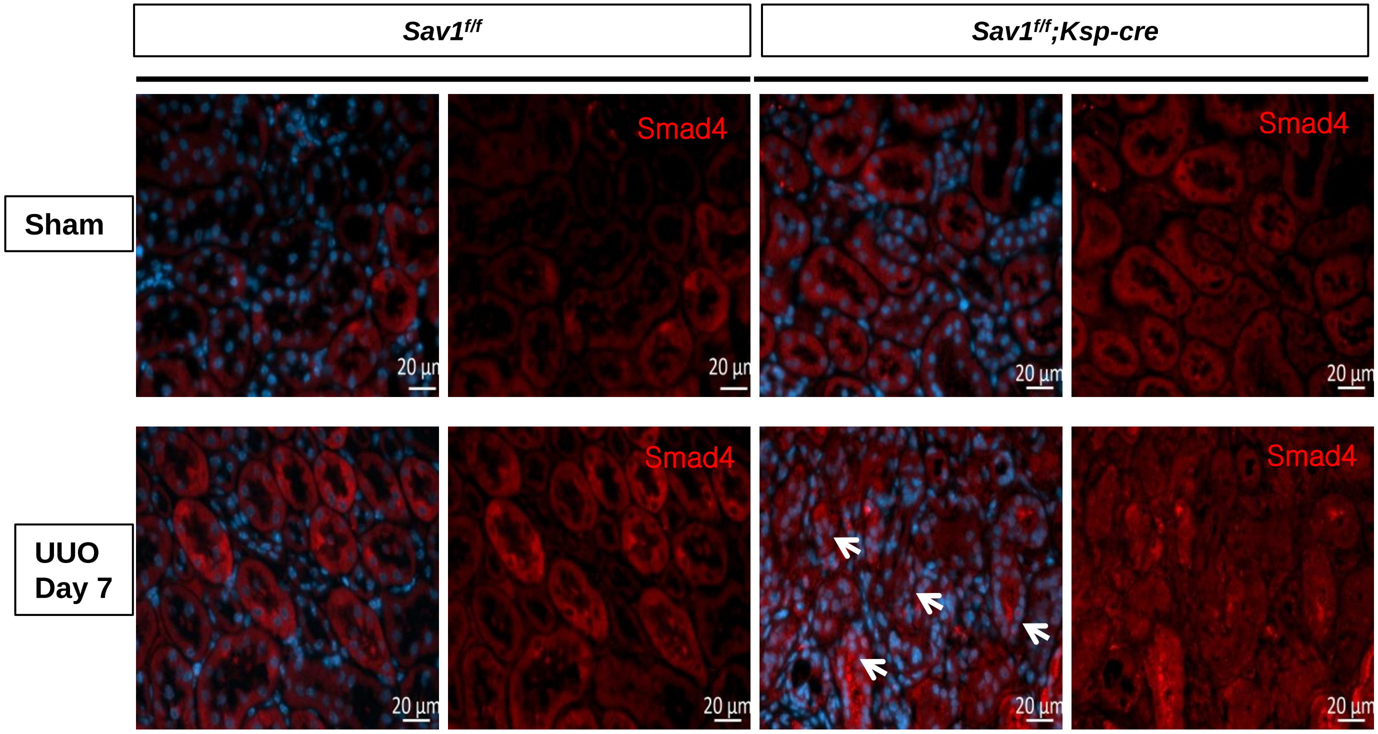

Supplementary figures 4

Oligos

Col1A1_F	ATCAACCGGAGGAATTTCCGT
Col1A1_R	CACCAGGACGACCAGGTTTTC
Col3A1_F	GCCAAATATGTGTCTGTGACTCA
Col3A1_R	GGGCGAGTAGGAGCAGTTG
aSMA_F	CTATGAGGGCTATGCCTTGCC
aSMA_R	GCTCAGCAGTAGTAACGAAGGA
CDH1_F	CGAGAGCTACACGTTCACGG
CDH1_R	GGGTGTCGAGGGAAAAATAGG
VIM_F	GACGCCATCAACACCGAGTT
VIM_R	CTTTGTCGTTGGTTAGCTGGT
SNAIL2_F	TGTGACAAGGAATATGTGAGCC
SNAIL2_R	TGAGCCCTCAGATTTGACCTG
NEGR1_F	AGAGTGAGCAGCCCTAGCAG
NEGR1_R	GGGAAGTCCACACTCTGTCC
hTGFbR2_F	GTAGCTCTGATGAGTGCAATGAC
hTGFRb2_R	CAGATATGGCAACTCCCAGTG
hTGFbRII_CHIP F	GCGCAAATGATTATGCAAGA
hTGFbRII_CHIP R	TTTGGTCAATTCACAACAGCTC
hTGFbRII_F (luciferase assay)	CATAAAAAATATCAAAGGCAACCTCT
hTGFbRII_R (luciferase assay)	CAACTCCAGAAGCTTTGTTAATTTG
Axin2_F	TGACTCTCCTTCCAGATCCCA
Axin2_R	TGCCCACACTAGGCTGACA
TGFb2_F	CCATCCCGCCCACTTTCTAC
TGFb2_R	AGCTCAATCCGTTGTTCAGGC
hTbRII_lF	CATAAAAAATATCAAAGGCAACCTCT
hTbRII_lR	CAACTCCAGAAGCTTTGTTAATTTG
mTGFbR2_F	CCGCTGCATATCGTCCTGTG
mTGFbR2_R	AGTGGATGGATGGTCCTATTACA
mTGFb2_F	TCGACATGGATCAGTTTATGCG
mTGFb2_R	CCCTGGTACTGTTGTAGATGGA
